# Fabrication of biosynthesized nickel ferrites nanoparticles and evaluation of their insecticidal efficacy on beetles (*Blaps polychresta*) testicular integrity

**DOI:** 10.1038/s41598-025-90496-0

**Published:** 2025-02-28

**Authors:** Esraa A. Arafat, Abdelazeem S. Eltaweil, Eman M. Abd El-Monaem, Hanan I. Elhenawy, Hussein K. Hussein, Lamia M. El-Samad, Mohamed A. Hassan

**Affiliations:** 1 Department of Zoology, Faculty of Science, Alexandria University, 21321 Alexandria, Egypt; 2 Department of Engineering, Faculty of Technology and Engineering, University of Technology and Applied Sciences, Ibra, Sultanate of Oman; 3 Chemistry Department, Faculty of Science, Alexandria University, Alexandria, Egypt; 4 Advanced Technology Innovation, Borg El-Arab, Alexandria, Egypt; 5 Protein Research Department, Genetic Engineering and Biotechnology Research Institute (GEBRI), City of Scientific Research and Technological Applications (SRTA- City), 21934 New Borg El-Arab City, Alexandria, Egypt

**Keywords:** Apoptosis, *Blaps polychresta*, DNA damage, Green synthesis, Nanoparticles, Nanopesticides, Nickel ferrites, Oxidative stress, Pest management, Testicular tissue, Ultrastructure, Darkling beetles, Zoology, Materials science, Nanoscience and technology

## Abstract

Green synthesis of nanoparticles has emerged as a significant strategy to develop effective and eco-friendly insecticide agents to combat insecticide resistance and preserve environmental integrity and biodiversity. This study was thus designed to fabricate novel green synthesized NiFe_2_O_4_ nanoparticles (NiFe NPs) and investigate their potential insecticidal effects for the first time using *Blaps polychresta* beetle as an agricultural coleopteran pest model. Therefore, we prepared NiFe NPs following the hydrothermal synthesis procedure in the presence of lemon juice. The physiochemical characteristics of NiFe NPs were investigated employing SEM, TEM, FT-IR, XRD, TGA, VSM, and UV-Vis analysis. The lowest and most effective dose of NiFe NPs against male beetles was ascertained at a concentration of 0.03 mg/g body weight, reporting 67% mortality after 48 h. To study the insecticidal impact of NiFe NPs, EDX analysis demonstrated the bioaccumulation of NiFe NPs in testicular tissues of beetles, leading to pathophysiological consequences. Precisely, the oxidative stress incited by NiFe NPs led to disturbance of the antioxidant defense system, which was defined by augmentation of lipid peroxidation and suppression of antioxidant enzymes. Furthermore, the comet assay exhibited remarkable DNA impairment, while flow cytometry analysis showed substantial cellular necrosis and apoptosis in NiFe NPs-treated beetles compared to control insects. In correlation with these findings, several aberrations in the histological and ultrastructure attributes of testicular tissues were perceived, including impaired follicular and cyst walls, deteriorated parietal cells, necrosis, and vacuolations. These results implied that NiFe NPs triggered oxidative injury in the testes, resulting in male reproductive system dysfunction. Altogether, our findings accentuate the potential application of NiFe NPs as nanopesticides, paving the way for the sustainable and cost-effective management of insect pests in agriculture.

## Introduction

Widely grown grain crops are rich sources of vitamins, carbohydrates, proteins, fats, minerals, and oils, making them highly prone to insect pest infestations, which represent about one-third of grown crop damage^1,2^. Feeding of these pests not only impairs the marketability of the grains but also makes them inadequate for human consumption^1^. The resurgence of insect pests due to increased climatic change and global warming further exacerbates the issue^3^. Chemical control is one of the most effective strategies to manage notorious insect pests, but its effectiveness is challenged by insecticide resistance and climate change. It has been reported that insecticide resistance has emerged in more than 550 insect pest species^4^. Additionally, chemical pesticides can substantially imperil the environment and public health with the growth of global agricultural production^5^. Therefore, many endeavors are now focused on designing sustainable and eco-friendly pest control strategies to curtail the challenges and jeopardies associated with chemical control^3^.

Nanotechnology has garnered significant interest in revolutionizing agriculture by introducing innovative and sustainable pest control approaches with high efficacy^6^. Nanoparticles (NPs) are a subcategory of nanomaterials with particle sizes ranging from 1 to 100 nm, bestowing them with exceptional physicochemical characteristics with versatile applications in various fields^7,8^. Therefore, different nanomaterial-based insecticides have been developed, including nanosuspensions, nanoemulsions, and solid-based nanopesticides, and for safeguarding crop products^1^. Interestingly, the small size of these formulations represents their major advantage in reducing the detrimental impacts on non-target species and contributing to their proper dispersal over the pest surface, resulting in improved action compared to conventional pesticides^1^. Among the applications of conventional nanopesticides, green synthesized nanoparticles have been recently implemented due to their high compatibility, specificity towards the target pest, and eco-friendly properties^9^. In addition, green synthesized nanopesticides can penetrate deep into pest inter- and intracellular spaces^6^.

Among the nanoparticles, ferrites are increasingly becoming a prominent class of nanomaterials that are broadly applied in various applications^10^. Nickel ferrites (NiFe_2_O_4_) find their way in multifarious sectors, including biomedical and environmental remedial applications, due to their distinctive characteristics among the ferrite family^10^. These characteristics include elevated electrochemical and mechanical stability, permeability, and electrical conductivity, in addition to the ability to resist corrosion^11^. Typically, NiFe NPs are prepared by diverse fabrication routes, comprising sol-gel, co-precipitation, hydro-thermal, and microwave-assisted^12,13^. Nevertheless, the bio-assisted or green fabrication approach demonstrates remarkable features over the other physical or chemical methods, where it is a safe and non-toxic process with cost-effectiveness merit and minimal environmental impact^14,15^.

It is believed that Coleopterans and Lepidopterans are the major storage insect pests, inflicting losses for stored grains with about 600 and 70 species, respectively^1^. Globally, Coleopterans are the largest and most diverse insect taxon^16^, nearly inhabiting all terrestrial environments, thus interacting with a wide range of organisms and performing major roles in numerous ecosystems^17^. Previous reports demonstrated the use of a wide variety of coleopteran species as bioindicators, while many of these species have the potential to contaminate stored products worldwide, leading to significant economic losses^17^. For instance, *Blaps polychresta* (Forskal, 1775), cosmopolitan Tenebrionid Coleopterans, have been considered of significant importance to ecosystems and have extreme resistance to various experimental treatments^18^. Therefore, there is a critical need for the investigation of novel insecticides and their effectiveness on beetles, considering their potential resistance.

The potential mechanism of nanopesticides can be attributed to the triggering of reactive oxygen species (ROS) overproduction, resulting in cellular injury through their interaction with lipids, proteins, and nucleic acids, leading to lipid peroxidation, membrane destruction, enzymatic dysfunction, DNA impairment, apoptosis, and necrosis^19^. It is assumed that insects can overcome nanoparticle intoxication and ROS elevation through various antioxidant and detoxification enzymes comprising superoxide dismutase (SOD), reduced glutathione (GSH), and β-carboxylesterase (CarE), respectively^20^.

It is acknowledged that conventional nanopesticides pose significant threats to both aquatic and terrestrial ecosystems due to their exceptional attributes, such as enhanced mobility and prolonged persistence^21^. Given the small size of nanopesticides, they are prone to quick degradation in sunlight and premature evaporation, which negatively affect their effectiveness^21^. Additionally, nanopesticides can accumulate in soil and water, affecting non-target organisms and disrupting ecosystems^22^. The toxicity of metallic nanopesticides to beneficial insects and their potential to enter the food chain raise further environmental and health issues^23^. Due to insects’ resistance to conventional nanopesticides, higher doses of nanopesticides are necessary, resulting in expanded environmental hazards^23^. Furthermore, this presents opportunities to enhance insect resistance through detoxification enzymes and modifications to their cuticle structure, thereby reducing pesticide infiltration. To alleviate these issues, sustainable, cost-effectiveness, and eco-friendly pest management approaches are needed^3,21,24^.

Therefore, green synthesized nanoparticles are getting profuse attention for their application as effective and safe alternatives to conventional nanopesticides globally. Herein, this study is the first report to provide in vivo application of biosynthesized NiFe NPs as nanopesticides using beetles as a model agricultural pest. We thus evaluated the lowest and most effective dose of the biosynthesized NiFe NPs utilizing *B. polychresta* beetles as a model insect. To unravel the insecticidal properties of the NiFe NPs, we investigate potential pathophysiological consequences, including oxidative stress parameters, cell viability, and DNA impairment through ensuring the bioaccumulation of NiFe NPs in testicular tissue using EDX analysis. Additionally, histological and ultrastructural attributes of the testicular tissues of beetles were examined to explore the anomalies in the tissue structure.

## Materials and methods

### Materials

Lemon was purchased from a shop in Alexandria, Egypt. Nickel chloride hexahydrate (NiCl_2_.6H_2_O) and sodium hydroxide (NaOH) were purchased form Alfa Aesar, while ferric chloride hexahydrate (FeCl_3_.6H_2_O) was provided by Alpha Chemika.

### Green synthesis of NiFe NPs

**Scheme 1:**
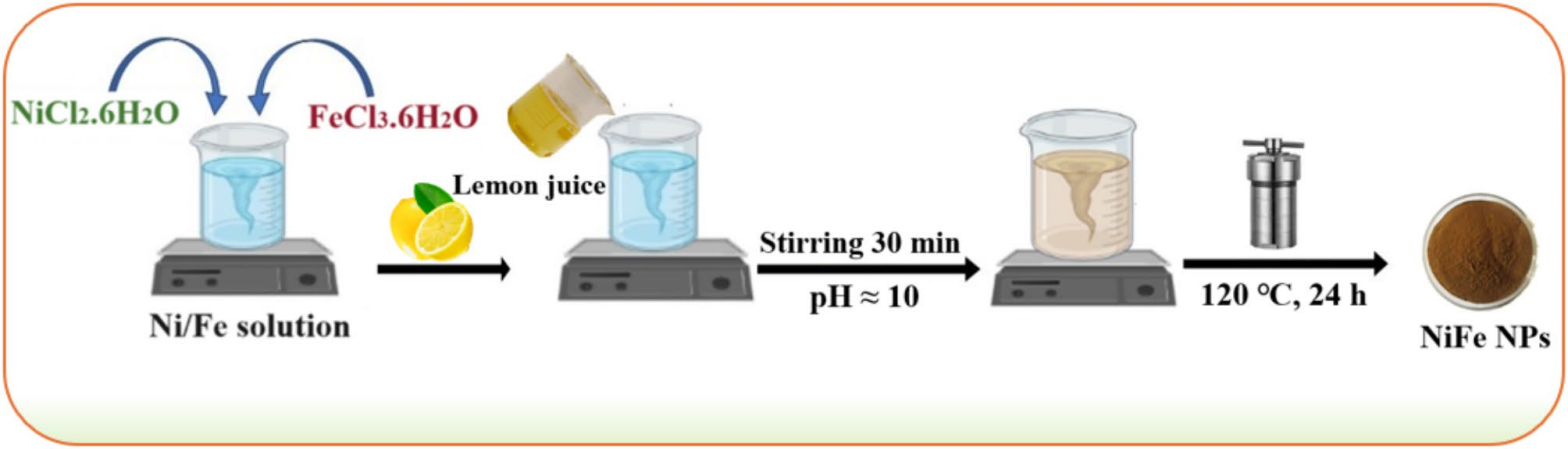
Hydrothermal synthesis of green NiFe NPs using lemon juice.

NiFe_2_O_4_ nanoparticles (NPs) were fabricated using a hydrothermal approach. Initially, 2.0 M of each NiCl_2_·6H_2_O and 4.0 M of FeCl_3_.6H_2_O were dissolved in 100 mL of double-distilled water under vigorous stirring to obtain a homogeneous Ni/Fe solution. Subsequently, 25 mL of lemon juice was added to the solution and the stirring continued for further 30 min. The pH of the solution was adjusted to 10 using aqueous NaOH (0.1 M). The resulting suspension was transferred to an autoclave and heated at 120 °C for 24 h. After cooling, the chocolate-brown NiFe_2_O_4_ NPs were washed and dried at 80 °C and donated as NiFe NPs. Scheme 1 illustrates the hydrothermal synthesis of green NiFe NPs.

### Characterization apparatus

Various characterization instruments were applied to investigate the chemical characteristics of the fabricated NiFe NPs. The outer morphology and the size of the NiFe NPs were investigated by scanning electron microscope (JEOL JSM-5300, Japan) and transmission electron microscope (JEM-1400 Plus, Japan). The chemical composition of NiFe NPs was investigated by Fourier Transform Infrared (FTIR, PerkinElmer, USA), and its crystallite phase was inspected using X-Ray Diffraction (XRD, PANalytical, UK). The thermal stability of the prepared NPs was evaluated by Thermal Gravimetric Analyzer (TGA, Shimadzu-50, Japan) and the Vibrating Sample Magnetometer (VSM, Lakeshore, USA) clarified the magnetic property of NiFe NPs. The well-formation of NiFe NPs was evinced by UV-Vis spectroscopy (UV-Vis, PerkinElmer, USA).

### Insect collection

Beetles were collected in two phases: the first phase was for evaluating survival and mortality analyses, and the second was for conducting further investigations in this study. Beetles were collected from the organic garden of the Faculty of Science, Moharam Bek, Alexandria University, Alexandria, Egypt, which was previously defined as a non-polluted site^7^. Beetles were identified as *Blaps polychresta* (Forskal, 1775) (Coleoptera, Tenebrionidae) and housed in the entomology lab at the Faculty of Science, Alexandria University, Egypt, within standard wood cages under sterilized conditions with unrestricted access to food and water. They kept for three days for the acclimatization with lab conditions (temperature: 29.4 ± 3.5 °C, humidity: 46.5 ± 9.4%, and photoperiod (light: dark of 12:12 h) before commencing the experiment.

### Experimental setup

The first step of this study was to evaluate the most effective dose of NiFe NPs on the mortality of adult male *B. polychresta* beetles. Thus, male beetles were divided randomly into six groups (12 beetles/group); the first group was the control group, and the other five groups were injected with five ascending doses of NiFe NPs at final doses of 0.01, 0.03, 0.05, 0.07, and 0.09 mg/g body weight, respectively. Beetles were anesthetized on ice for 5 min before being punctured in the caudal abdominal sternites by means of 1 mL BD hypodermic syringe (27-gauge and 1/2-inch needle). The needle remained in a horizontal position to prevent any unintended interior damage. The mortality rate of male beetles was reported after 24 h of exposure to NiFe NPs and observed the insects daily for 10 days. The mortality and survival rates were analyzed following Kaplan-Meier analysis. According to mortality evaluations of adult male *B. polychresta*, 240 male beetles were divided randomly into two groups (120 beetles/group), representing the NiFe NPs-treated group that was injected once with NiFe NPs at a dose of 0.03 mg/g body weight and the control group, which was injected with an equivalent amount of normal saline solution.

### Dissection procedure of testicular tissues

After five days of injecting male beetles with NiFe NPs or saline solution, they were dissected as previously described^25^. Briefly, beetles were euthanized on ice for 10 min and then dissected using a stereomicroscope through the lateral opening in the abdominal area. Afterward, testicular tissues were removed and placed either in 4% formaldehyde:1% glutaraldehyde fixative (pH 7.2) for ultrastructural investigations or preserved at − 80 °C for biochemical and molecular assays.

### Determination of NiFe NPs accumulation in testicular tissues of *B. polychresta*

Using a scanning electron microscope connected with an energy dispersive X-ray micro-analyzer (SEM-EDX) (JEOL JSM-5300, Japan) at 20 kV accelerating voltage, NiFe NPs accumulated in adult *B. polychresta* testicular tissues were evaluated. For these investigations, we examined five randomized specimens from each group, and mass percentages were calculated employing EDX in relation to reference elements.

### Biochemical assessments in testicular tissues of *B. polychresta*

Lipid peroxidation was appraised by evaluating the malondialdehyde (MDA) levels according to the previously published procedures^26^. The reaction was conducted based on the reaction of MDA found in testicular homogenate with thiobarbituric acid (TBA), resulting in a pink product, and the absorbance was measured at 532 nm. Furthermore, the enzymatic activity of β-carboxylesterase (CarE) was assessed as reported earlier^27^, while the superoxide dismutase (SOD) and reduced glutathione (GSH) activities were evaluated utilizing the EnzyChromTM superoxide dismutase assay kit (ESOD-100, BioAssay Systems, USA) and reduced glutathione kit (Elabscience, USA), respectively, according to the manufacturer’s procedures. All measurements were conducted from 3 to 5 replicates.

### Measurement of mRNA expression of HSP70 and MT1

The total RNA was isolated from the testicular tissues of the control and NiFe NPs-treated groups *B. polychresta* employing TRIzol™ Plus RNA Purification Kit (Invitrogen, Cat. No. 12183555, USA) following the manufacturer instructions. The quality and quantity of the purified RNA were determined using UV absorbance at 260 nm, and its integrity was then examined utilizing agarose gel electrophoresis. The High-Capacity cDNA Reverse Transcription Kit (Cat. No. 4374967, Applied Biosystems, USA) was applied to perform total RNA reverse transcription. Two-step real-time RT-PCR using Maxima SYBR Green qPCR Master Mix (2X) (Applied Biosystems, USA) was executed to analyze the relative gene expression levels of HSP70 and MT1 in the presence of an HSP70 forward primer (5`-TGGCGGCAAACCGAAGAT-3`), HSP70 reverse primer (5`-CGCTGGCACCGTAATGAC-3`), MT1 forward primer (5`-GTTGCTGAAGCCGCCTACT-3`), MT1 reverse primer (5`-CATCTTGGGTGGCTGGTG-3`), along with β-actin gene as control with forward primer (5`-CTCTGCTATGTAGCCCTTGACTT-3`), and reverse primer (5`-GCAGTTGTAGGTGGTTTCGTG-3`). The two-step cycling protocol for each gene was programmed as follows: the first step was one cycle at 50 °C for 2 min, the second one was one cycle at 95 °C for 10 min, then 40 cycles of 95 °C for 15 s and 60 °C for 60 s. The RT-qPCR reactions were replicated thrice for all genes and the 2^−ΔΔCt^ equation was applied to calculate mRNA expression as reported earlier^28,29^.

### Genotoxicity assessment

A comet assay was used to assess the level of DNA impairment as a result of beetles’ exposure to NiFe NPs compared to the control beetles. Testicular tissues were randomly selected from each group to conduct this test, and the analysis was conducted according to a previously published approach^30,32^. To ensure high accuracy, three slides from each group were prepared, and 100 cells per slide were investigated. Accordingly, five parameters were determined, including the comet tail length, tail moment, percentage of DNA in the comet tail, and the percentage of tailed and untailed cells.

### Cytotoxicity measurement

To detect apoptosis and viability in testicular cells obtained from NiFe NP-treated beetles compared to control beetles, the Annexin-V-FITC/PI staining kit (Cat. No. TA4638, Sigma-Aldrich, Germany) was utilized, and the analysis was performed by means of flow cytometric analysis following manufacturer guidelines, as previously described^7^. The results were analyzed employing flow cytometry Cell Quest Pro software (5.2.1, 2005, Becton Dickinson, USA).

### Histological and ultrastructural investigations of *B. polychresta* testicular tissues

Testicular tissues fixed in 4 F:1G as described above were used for pathohistological and ultrastructural investigations. Following a wash with 0.1 M phosphate buffer, the specimens were dehydrated by ascending ethanol concentrations. Afterward, the tissues were embedded in an Epon-Araldite blend (Sigma Aldrich, France), followed by slicing at a thickness of 0.5 μm with LKB-ultramicrotome (LKB Bromma 2088 Ultrotome, Leica Instruments, Bannockburn, USA) for semithin section preparation^33^. Semithin sections were mounted and stained with toluidine blue and then surveyed using a light microscope (Olympus CX31, Japan). Ultrathin sections of 60 nm thick were prepared, mounted on copper grids (200 mesh), stained twice for 30 min each with uranyl acetate and lead citrate, and examined at an acceleration voltage of 80 kV with a transmission electron microscope (TEM, JEM − 1400 Plus, Tokyo, Japan)^34^.

For SEM examination, after fixation of the tissues, they were postfixed for 2 h in 2% osmium tetroxide at 4 °C, washed in PBS, and dehydrated employing a critical point dryer (Minnesota, USA). Following this, the samples were mounted on aluminum-stub, followed by coating with a gold-palladium sputter coater (JFC-1100 E). SEM at 20 kV accelerating voltage (JEOL JSM-5300, Japan) was used for inspecting the tissues.

### Statistical analysis

All experiments performed in this study were conducted in 3–5 replicates, and statistical analysis was carried out utilizing SPSS (Version 25, IBM Software, Inc., Chicago, IL, USA) and GraphPad Prism (Version 8, GraphPad Software Inc., San Diego, CA, USA). To analyze mortality, Kaplan-Meier survival analysis was carried out with a log-rank (Mantel-Cox) test, and the Chi-square test was calculated. To ensure the validity of the results, the normal (Gaussian) distribution of the data was assessed using the Shapiro-Wilk test. All results were then analyzed with a two-tailed unpaired Student’s t-test to assess the significant difference between the control and NiFe NPs-treated groups. The significant difference was determined at *p ≤ 0.05, whereas the high significant differences were determined at **p ≤ 0.01, ***p ≤ 0.001, and ****p ≤ 0.0001. All figures illustrated in this study were plotted, presenting all results as mean ± SD.

## Results

### Characterization of NiFe NPs

#### FTIR

**Fig. 1:**
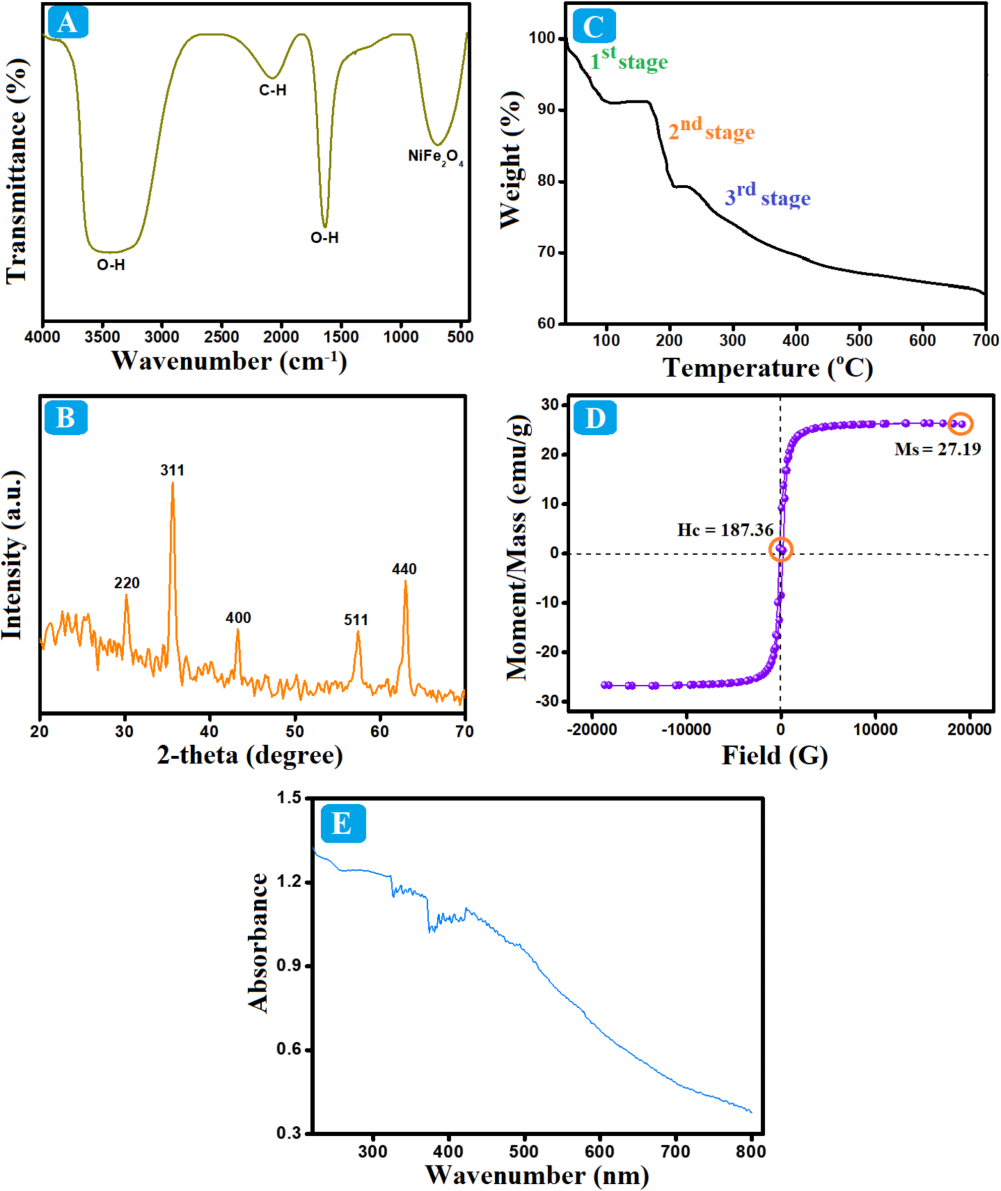
**(A)** FTIR, **(B)** XRD, **(C)** TGA, **(D)** VSM, and **(E)** UV-Vis of NiFe NPs.

The chemical functional groups of NiFe NPs were defined using FTIR analysis, as illustrated in Fig. 1A. The FTIR peak at 691 cm^− 1^ is allocated to the octahedral NiFe NPs, confirming their successful fabrication. The distinct band of the bending OH group manifested at 1634 cm^− 1^, and the broadband at 3435 cm^− 1^ is assigned to the stretching vibration of OH of the adsorbed water. This existing OH group facilitates the conjugation and dispersion of NiFe NPs. The observed peak at 2078 cm^− 1^ is ascribed to aromatic C-H in the limonene structure.

#### XRD

The crystallographic phase of NiFe NPs was scrutinized by XRD (Fig. 1B). The XRD pattern depicted diffraction peaks at 2-theta 30.22°, 35.54°, 43.17°, 57.35°, and 62.92°, which are related to planes of 220, 311, 400, 511, and 440, sequentially. This XRD pattern denoted the cubic spinel structure of NiFe NPs, matching with the JCPDS 74-2081 card.

#### TGA

The thermal performance of NiFe NPs was investigated by TGA (Fig. 1C). The thermogram illustrates three stages of weight loss; the 1st stage at the temperature range 36–190 °C corresponds to the evaporation of the adsorbed solvent. The 2nd stage started at a temperature of 190 °C and ended at 227 °C, which is related to the organic materials oxidation of the limonene structure. Furthermore, the 3rd stage between 227 and 452 °C owes to the metal oxide formation. Beyond 452 °C there was no considerable weight loss, which is most probably due to the growth of the nano-crystals of NiFe NPs.

#### VSM

The magnetization of NiFe NPs was studied utilizing VSM (Fig. 1D). The magnetism hysteresis loop demonstrated the ferromagnetic character of NiFe NPs, where the coercivity was about 187.36 G. Furthermore, the value of saturation magnetization was 27.19 emu/g, denoting the potent magnetic property of NiFe NPs that endows it the easy separation feature via a magnet.

#### UV-Vis spectroscopy

NiFe NPs were analyzed using at a wavelength range of 200–800 nm (Fig. 1E). The UV-Vis graph of NiFe NPs elucidates a peak at 341 nm, suggesting the formation of nanoparticles of NiFe NPs with a nano size less than 20 nm. Generally, the increase in particle size results in red shifting of the UV-Vis peaks owes to occurring light scattering. Hence, the manifestation of peaks at 386, 393, and 399 nm indicated the presence of NiFe NPs in a size larger than 20 nm.

#### SEM and TEM

**Fig. 2:**
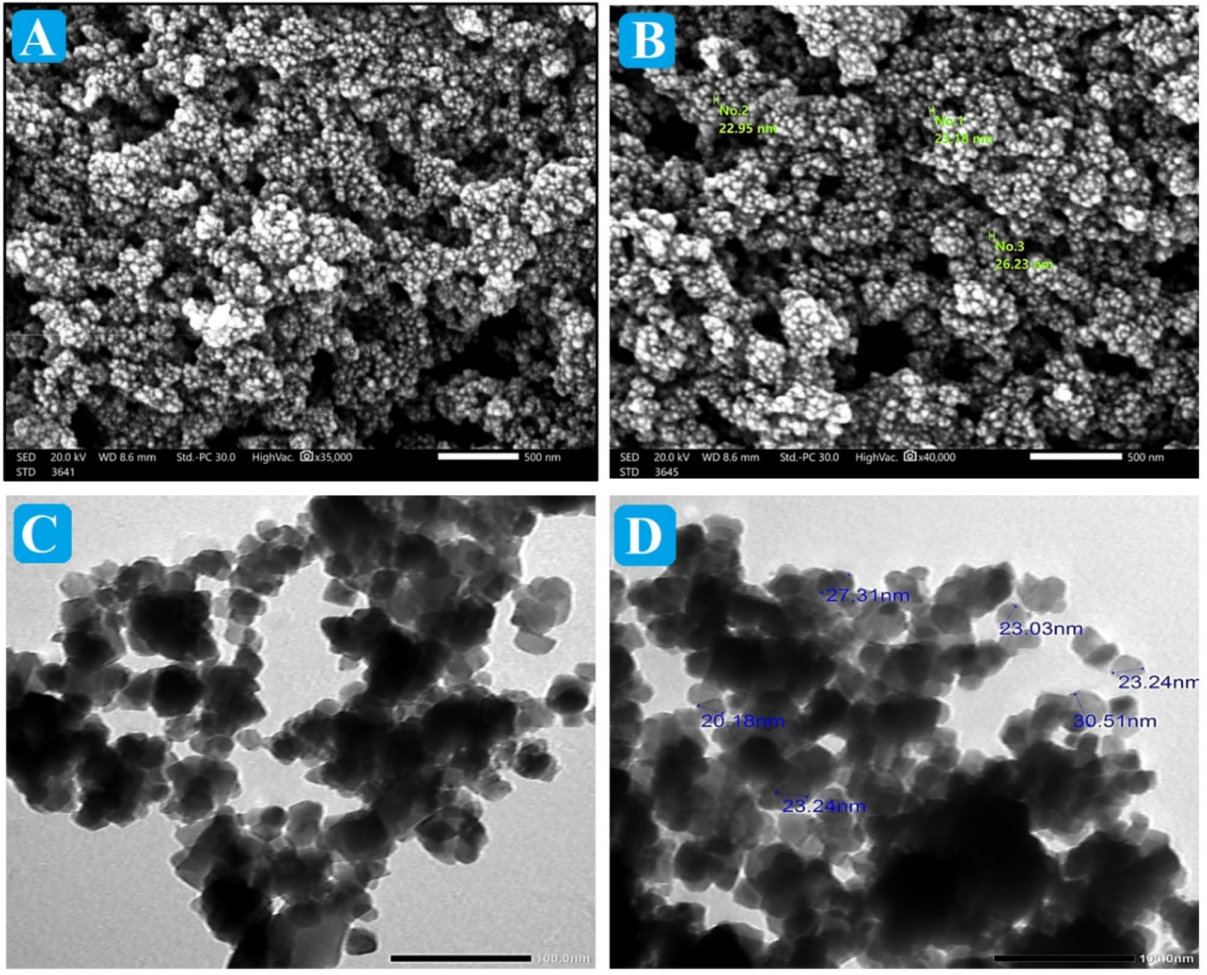
**(A and B)** SEM and **(C and D)** TEM images of biosynthesized NiFe NPs.

SEM was applied to define the outer morphology of NiFe NPs and their mean particle size (Fig. 2A and B). The SEM images showed semi-spheroidal particles of NiFe NPs that clumped together to form aggregation. Interestingly, it was found that the mean particle size of NiFe NPs was about 23.45 nm. Such a tiny particle size implied the high surface area of NiFe NPs. The TEM images revealed clusters of irregular particles of NiFe NPs and the small average sizes between 20.18 and 30.25 nm as presented in Fig. 2C and D. The aggregation in the SEM and TEM of NiFe NPs suggests the strong magnetic nature of NiFe NPs as depicted by VSM analysis^35^. In addition, the NiFe particles may be held together by external interactions like Van der Waals forces^36^.

### Mortality and survival analyses of *B. polychresta* after exposure to NiFe NPs

**Fig. 3:**
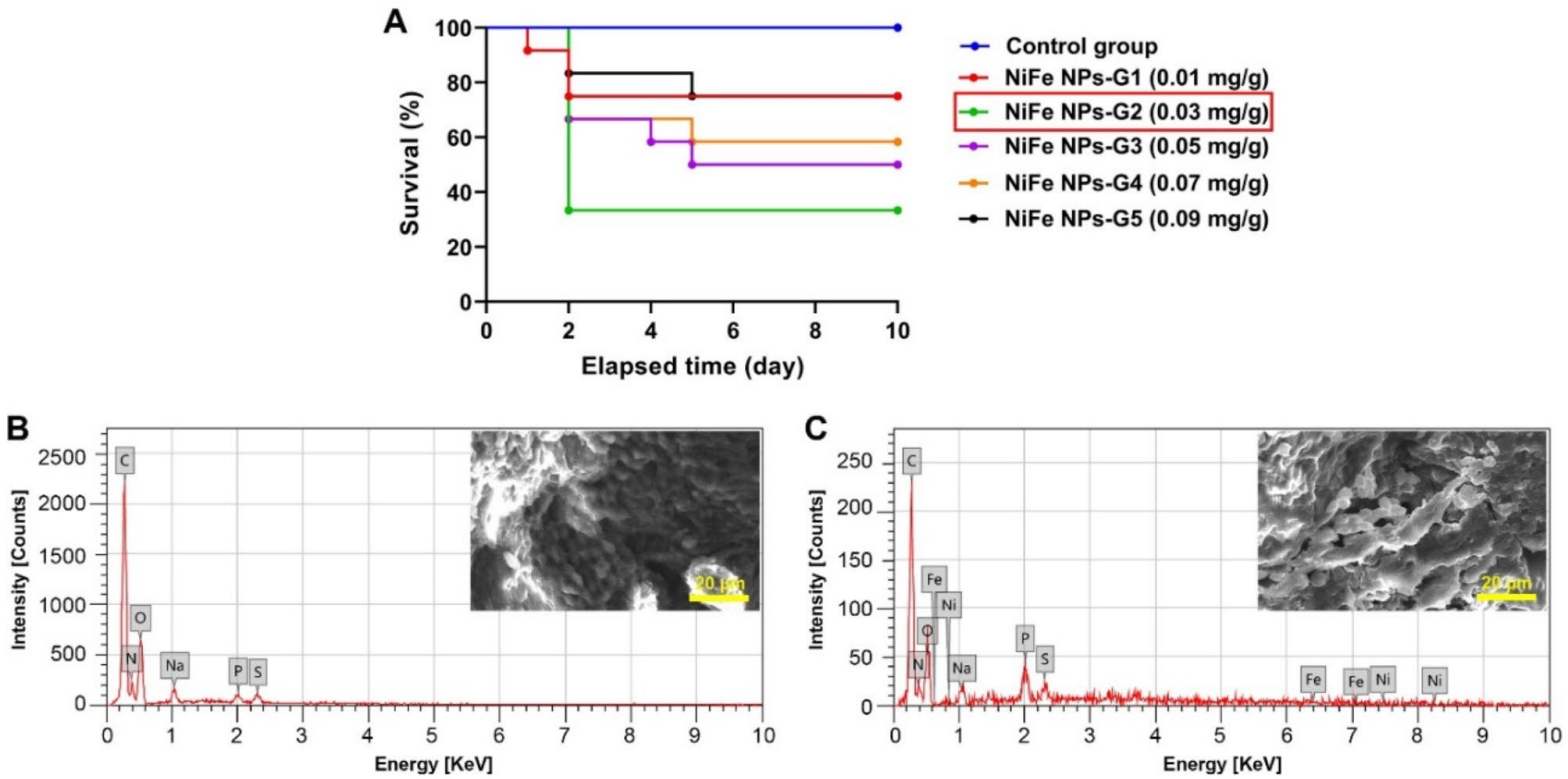
**(A)** Kaplan-Meier survival analysis showing the survival percentage of male *B. polychresta* for 10 days following exposure to NiFe NPs at different doses from 0.01 to 0.09 mg/g body weight and the Chi square was 13.22 at *p* < 0.05. **(B) and (C)** EDX spectra of the testicular tissues dissected from the control insects and those exposed to 0.03 mg/g body weight of NiFe NPs, respectively.

Five groups of the beetles were injected with five doses of NiFe NPs and monitored for ten days alongside the control insects. It could be extrapolated from Kaplan-Meier analysis that the dose of 0.03 mg/g body is the most effective and instant dose on mortality following exposure to NiFe NPs as shown in Fig. 3A. The results manifested the mortality effectiveness of NiFe NPs at a dose of 0.03 mg/g body weight on the second day of injection. This implies that this dose triggered deleterious effects on the beetles, leading to death. Therefore, further investigations, including biochemical, molecular, and ultrastructural studies were conducted using the testicular tissues dissected from the group of beetles injected with 0.03 mg/g body weight and those from the control beetles.

### Determination of NiFe NPs accumulated in testicular tissues of *B. polychresta*

As depicted in Fig. 3A and B, SEM-EDX analysis of testicular tissues obtained from NiFe NPs-treated insects revealed the accumulation of Ni and Fe compared to the control insects. It could be detectable from the EDX spectrum of the testicular tissues of control beetles the frequency of six elements, including carbon (C), nitrogen (N), oxygen (O), sodium (Na), phosphorus (P), and sulfur (S) as illustrated in Fig. 3B. On the other hand, similar elements were found in testes from the group of beetles subjected to NiFe NPs with a substantial accumulation of Ni and Fe as portrayed in Fig. 3C. Additionally, Table 1 presents the ratio of Ni and Fe accumulated in testes from the NiFe NPs-exposed group, reporting 0.21 ± 0.10% and 0.46 ± 0.21%, respectively.

**Table 1:** EDX analysis of testicular tissues from *B. polychresta* in response to NiFe NPs exposure.

Element	Control group	NiFe NPs-treated group
	Mass (%)	Mass (%)
C	47.46 ± 0.22^a^	50.52 ± 0.76^b^
N	18.54 ± 0.48^a^	15.49 ± 1.43^b^
O	31.27 ± 0.49^a^	28.24 ± 1.38^b^
Na	1.46 ± 0.07^a^	1.37 ± 0.20^a^
P	0.62 ± 0.04^a^	2.52 ± 0.20^b^
S	0.65 ± 0.03^a^	1.19 ± 0.14^b^
Fe	—	0.21 ± 0.10^b^
Ni	—	0.46 ± 0.21^b^

Values are shown as mean ± SD (*n* = 3). Different letters indicate significant differences between the control and NiFe NPs-treated groups at *p* < 0.05. (—) indicates not detected in midgut tissues.

### Impact of NiFe NPs application on the oxidative stress parameters of *B. polychresta*

**Fig. 4:**
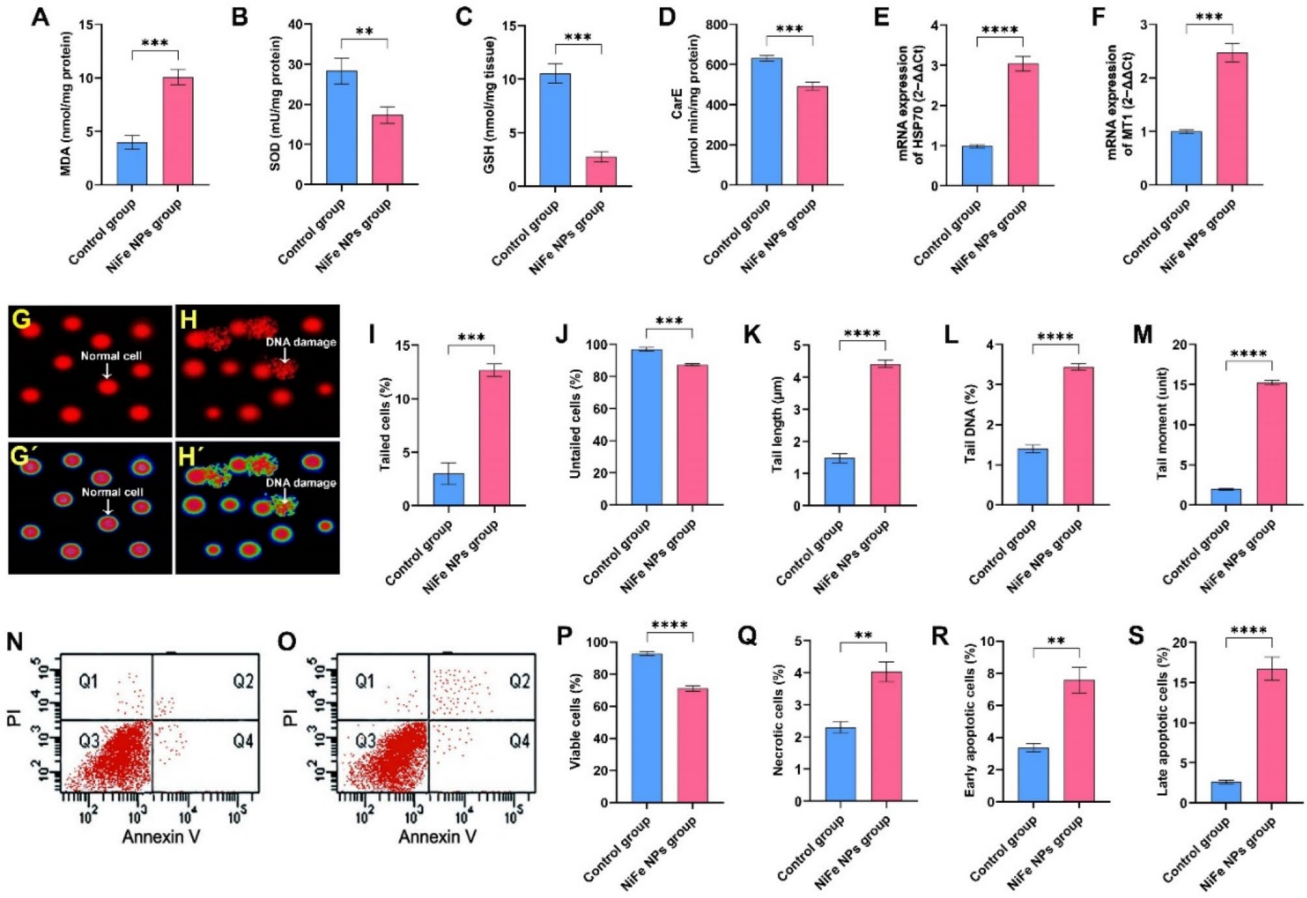
Assessments of (A) malondialdehyde (MDA), (B) superoxide dismutase (SOD), (C) reduced glutathione (GSH), (D) β-carboxylesterase (CarE), and relative mRNA expression of (E) HSP70 and (F) MT1 in *B. polychresta* testicular tissues obtained from the control and NiFe NPs groups. (G and H) and (G` and H`) images of comet assay analysis from the control and NiFe NPs-treated beetles, respectively. Comet assay analysis revealed (I) tailed cells (%), (J) untailed cells (%), (K) tail length (µm), (L) tail DNA (%), and (M) tail moment (unit). (N) and (S) Images for the Annexin-V/PI assay of the cells from the testicular tissues of the control and NiFe NPs-treated beetles, respectively. Q1, Q2, Q3, and Q4 refer to necrotic, late apoptotic, living, and early apoptotic cells, respectively. Flow cytometry analysis exhibited ratios of (P) viable cells, (Q) necrotic cells, (R) early apoptotic cells, and (S) late apoptotic cells. Results are shown as mean ± SD (****p < 0.0001, ***p < 0.001, and **p < 0.01).

In comparison to the control beetles, the group exposed to NiFe NPs exhibited a significant augmentation of the MDA level with significant difference (*p* < 0.001). Remarkably, the MDA concentrations in the testicular tissues of NiFe NPs-treated insects were almost two times greater than the MDA concentrations determined for the control group as displayed in Fig. 4A. The increase in MDA of NiFe NPs-treated insects was correlated with a substantial reduction in GSH level along with SOD and CarE activities compared to the control beetles with significant difference (*p* < 0.001, *p* < 0.01, and *p* < 0.001), respectively as demonstrated in Fig. 4B-D.

Additionally, assessments of mRNA expressions of HSP70 and MT1 exhibit that the treatment of insects with NiFe NPs gave rise to considerable upregulations of HSP70 and MT1 genes in testicular tissues compared to the control insects with significant difference (*p* < 0.0001 and *p* < 0.001), respectively as delineated in Fig. 4E and F. Notably, the testicular tissues of beetles treated with NiFe NPs showed a significant two-fold upregulation of MT1 compared to the control insects. Taken together, these results clearly indicated that the exposure of *B. polychresta* to NiFe NPs resulted in interference of antioxidant parameters, inciting oxidative stress in the testicular tissue.

### Genotoxicity evaluation

Genotoxicity evaluation of NiFe NPs against male *B. polychresta* testicular tissue compared to the control group was evaluated by comet assay as illustrated in Fig. 4G and H`. It is evident from the data in Fig. 4I and M that tail length, tail moment, percentage of tailed DNA, and percentage of tailed cells were noticeably higher in the beetles exposed to NiFe NPs than those detected in the control group with significant difference (*p* < 0.0001). On the other hand, the percentage of untailed cells was significantly lower in the NiFe NPs-treated group compared to the control insects with significant difference (*p* < 0.001). Collectively, these findings highlight the genotoxic influence of NiFe NPs on *B. polychresta* testicular tissue.

### Flow cytometry analysis

Cytotoxic properties of NiFe NPs against male *B. polychresta* testicular tissue in comparison with the control beetles were estimated using the annexin V-FITC assay as demonstrated in Fig. 4N and O. Precisely, Fig. 4P illustrates a significant reduction of 71 ± 1.7% in the viable cells of the beetles exposed to NiFe NPs, compared to 92.7 ± 1.3% in the testes of the control insects with significant difference (*p* < 0.0001). As shown in Fig. 4Q, this result correlates with a significant amplification of necrotic cells in the beetles exposed to NiFe NPs compared to the control group, representing 4 ± 0.3% and 2.3 ± 0.2%, respectively, with significant difference (*p* < 0.01). In a similar manner, early and late apoptotic cells exhibited a substantial augmentation in NiFe NPs-treated insects compared with the control beetles. In the testes of control battles, early and late apoptotic cells reported 3.3 ± 0.3% and 2.6 ± 0.2%, respectively, while these cells recorded 7.5 ± 0.8% and 16.7 ± 1.5%, respectively, in NiFe NPs-treated beetles with significant difference between control and treated beetles for both parameters (*p* < 0.01 and *p* < 0.0001), respectively (Fig. 4R and S). Overall, exposure to NiFe NPs elicited cellular toxicity in the testicular tissues of *B. polychresta*.

### SEM investigation

**Fig. 5:**
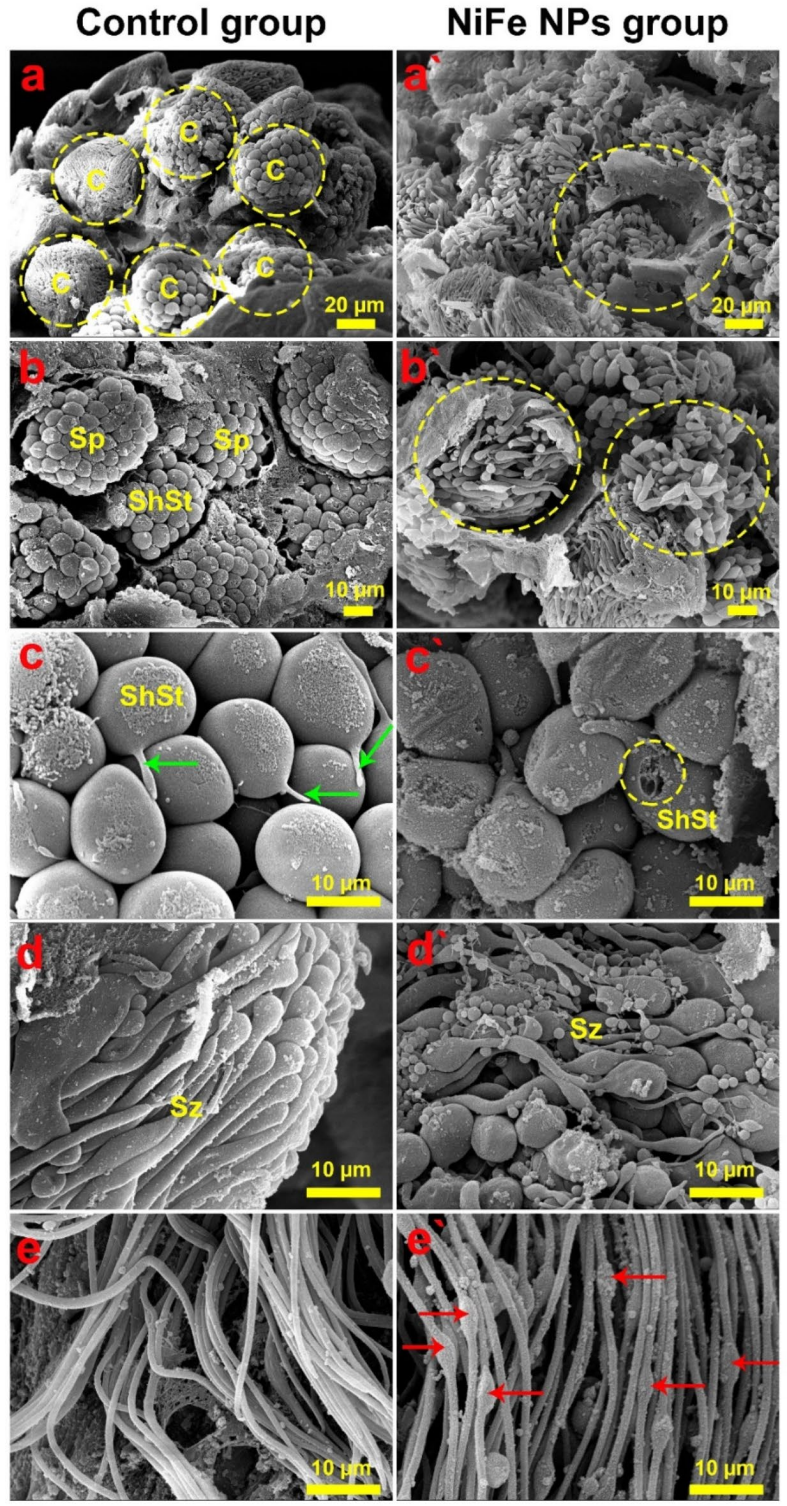
Scanning electron micrographs demonstrate the testicular morphology of *B. polychresta* dissected from the control (a-e) and NiFe NPs-exposed group (a`-e`). SEM examinations revealed that spermatogenic elements of diverse developmental stages occupied various cysts in the testicular follicles of the control beetles. (a) The SEM micrograph illustrates cysts (c) of different spermatogenic elements (encircled). Fig. (b) shows cysts filled with typical spermatocytes (Sp) and short spermatids (ShSt). Fig. (c) exhibits short spermatids (ShSt) and flagella (green arrows) with higher magnification. Fig. (d and e) depict the differentiation zone of *B. polychresta* dissected from control and NiFe NPs-treated insects. Fig. (d) delineates the normal morphology of spermatozoa (Sz). Fig. (e) displays the normal morphology of flagella. Fig. (a`) SEM micrographs from the NiFe NPs-treated group show a testicular follicle with undistinguished cysts (encircled). Fig. (b`) shows cysts with morphologically altered spermatogenic elements (encircled). Fig. (c`) represents abnormal short spermatids (ShSt) with ruptured heads (encircled) at a higher magnification. Fig. (d`) portrays anomalous differentiating spermatozoa (Sz), while Fig. (e`) represents atypical flagella morphology with signs of swelling (red arrows).

SEM examinations revealed that spermatogenic elements of diverse developmental stages occupied various cysts in the testicular follicles of the control beetles, implying a distinct global organization of normal spermatogenesis and spermiogenesis, as depicted in Fig. 5a and b. Accordingly, normal spermatocytes and short spermatids were perceived within the cysts associating with short spermatids with their round heads and short flagella as illustrated in Fig. 5c. Conversely, SEM analyses of the testicular tissues from the beetles exposed to NiFe NPs showed severe morphological aberrations throughout the different developing stages. It could be discernible that disordered cysts were observed within the testicular follicle, with hardly recognition of a single cyst, signifying that all spermatogenic elements were disrupted. This corroborates that the destruction of the cyst walls could be explained by the impairment of the parietal cells (Fig. 5a` and b`).

Additionally, Fig. 5c` delineate the morphologically altered spermatogenic elements, including ruptured short spermatids. Moreover, the differentiation zone of the control group was discerned with normal spermatozoa morphology (Fig. 5d), while Fig. 5e showed that the differentiating spermatozoa exhibited normal flagellar morphology. However, anomalous differentiating spermatozoa were detected in the testicular tissues treated with NiFe NPs (Fig. 5d`), which led to atypical morphology of flagella with manifestation of swelling (Fig. 5e`).

### Histological investigation

**Fig. 6:**
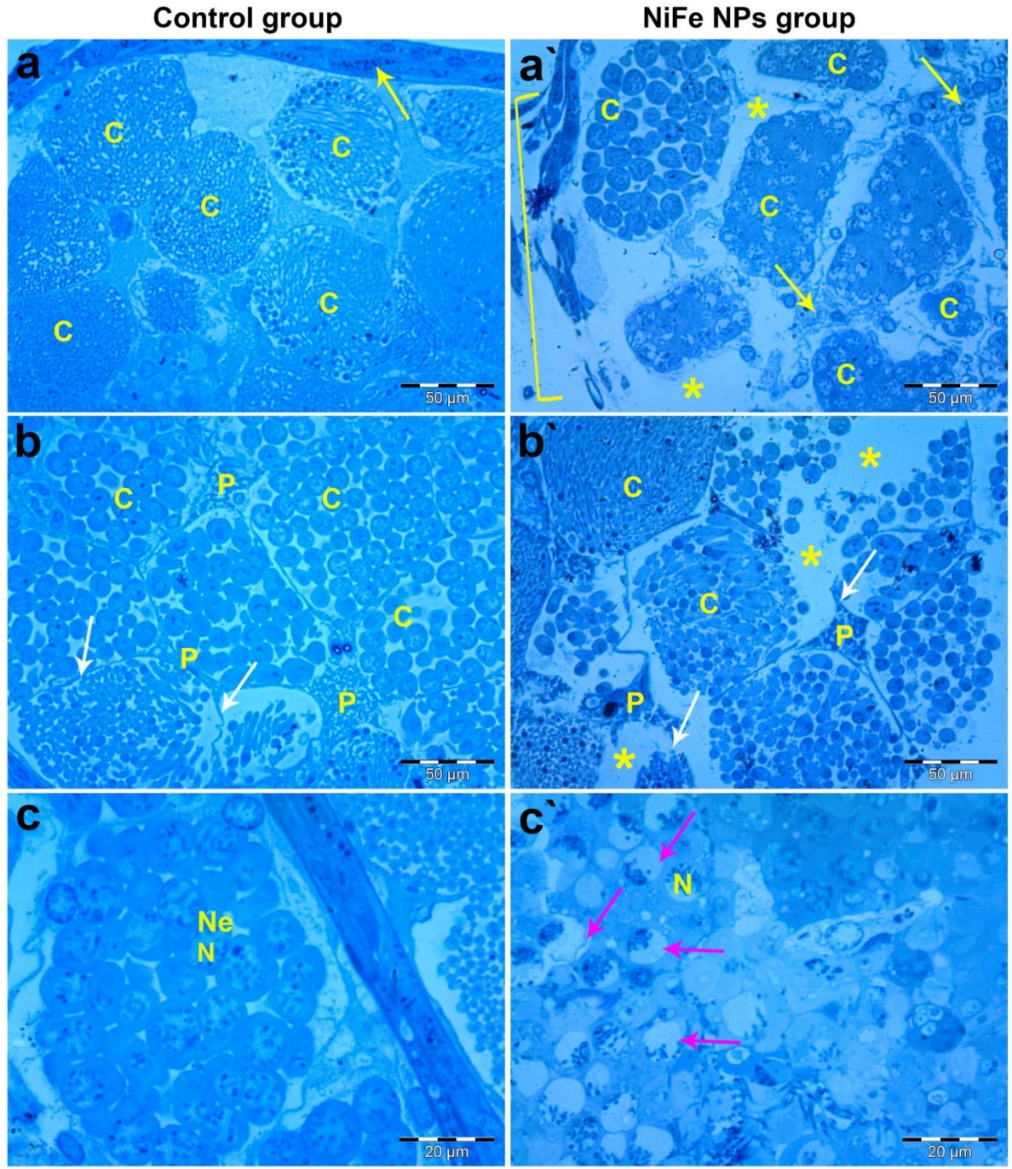
Semithin cross sections through the testicular tissue of adult male *B. polychresta* dissected from the control and NiFe NP-exposed groups. Histopathological investigations of the testicular cross-sections of adult *B. polychresta* from the control group revealed typical cysts with different stages of developing spermatogenic elements. (a-c) Light photomicrographs reveal the histological characteristics of the control beetles. Fig. (a and b) illustrate the intact follicular wall (arrow), various cysts (c) with typical cyst walls (white arrows), and parietal cells (P). Fig. (c) shows spermatogenic cells with obvious plasma membranes, apparent nuclei (N), and nuclear envelopes (Ne). Fig. (a`-c`) light photomicrographs illustrate the histopathological response after exposure to NiFe NPs. Fig. (a’ and b`) demonstrate the deteriorated follicular wall (bracket), necrosis (arrows), vacuolations (asterisk), ruptured cyst walls (white arrows), abnormal cysts (c), and parietal cells (P). Fig. (c`) reveals spermatogenic cells with severe karyorrhexis (arrows).

Histopathological investigations of the testicular cross-sections of adult *B. polychresta* from the control group revealed typical cysts with different stages of developing spermatogenic elements. Each cyst was characterized by an intact wall formed by the distinctive cytoplasmic extensions of the parietal cells and filled with well-organized cells within each cyst as illustrated in Fig. 6a and b. Furthermore, it is apparent from Fig. 6c that spermatogenic cells emerged with obvious plasma membranes and obvious nuclei and nuclear envelopes.

By contrast, remarkable histological alterations were discerned in the testicular tissues of *B. polychresta* as a result of exposure to NiFe NPs compared to the control group, including a lower number of cysts within the testicular follicles, deteriorated follicular walls, and ruptured cyst walls, which stem from the damaged cytoplasmic extensions of the parietal cells (Fig. 6a`&b`). Additionally, necrotic signs combined with vacuolations were noticed along the cysts, resulting in the loss of the regular organization of the spermatogenic elements and their synchrony. Also, disintegrated cells could be perceived with severe karyorrhexis as portrayed in Fig. 6c`.

### Ultrastructural investigation

**Fig. 7:**
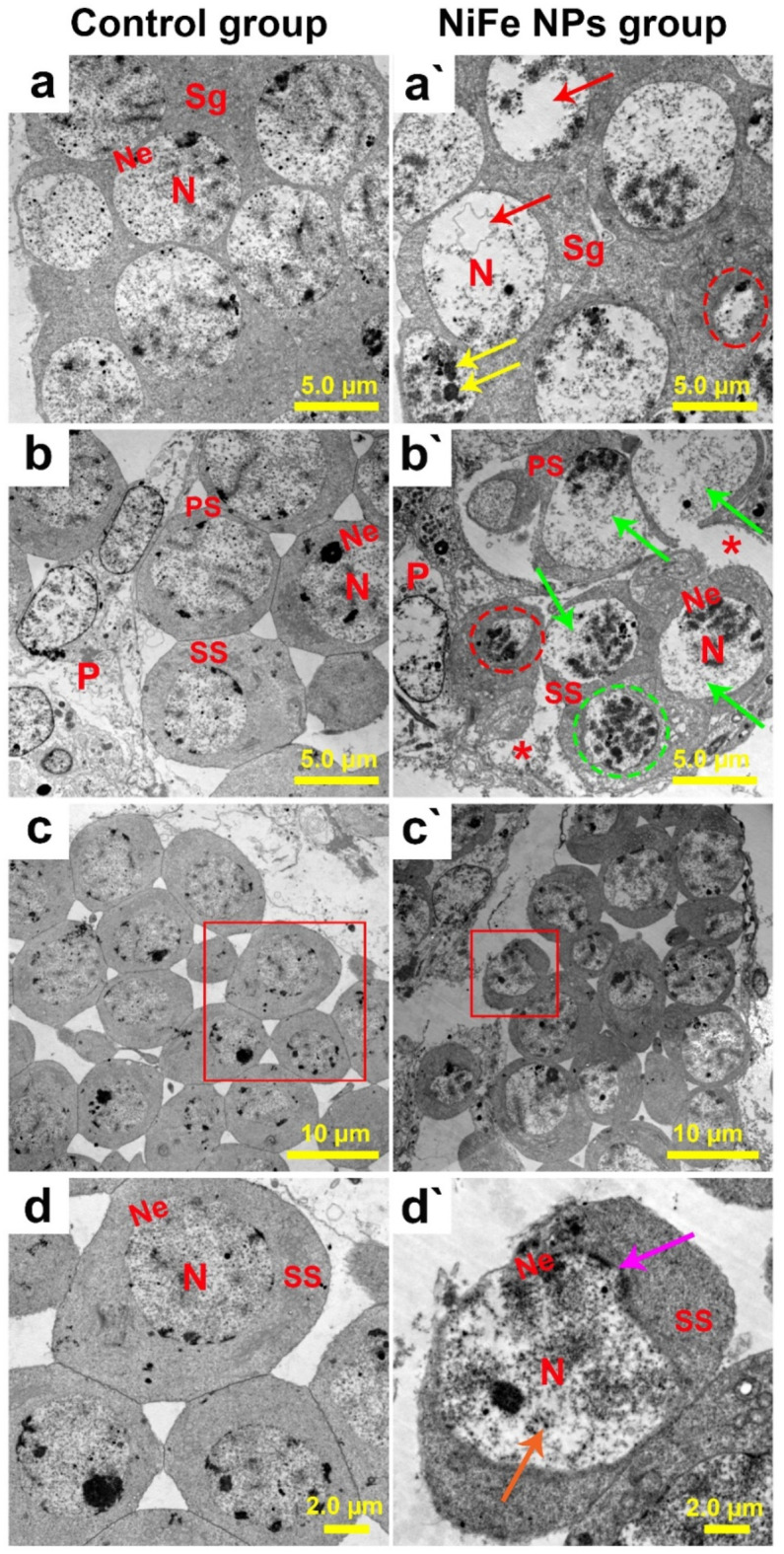
Ultrastructure analysis of the testicular growth zone of adult male *B. polychresta* dissected from the control (a-d) and NiFe NPs-exposed groups (a’-d`). Three different zones of development, involving growth, maturation, and differentiation was inspected. Fig. (a) shows the spermatogonia (Sg) with their large, round, homochromatic nuclei (N) and apparent nuclear envelopes (Ne). Fig. (b) illustrates normal primary spermatocytes (PS), secondary spermatocytes (SS), and parietal cells (P). Fig. (c) depicts typical secondary spermatocytes (SS). Fig. (d) shows a higher magnification of the inset in (c), centering on the nuclei (N) and their distinct nuclear envelopes (Ne). (a`) The TEM micrograph of the testis from the NiFe NPs-treated group shows pyknotic nuclei (encircled), electron-dense vesicles within the nucleoplasm (yellow arrows), and lysed and vacuolated nucleoplasm (red arrows). Fig. (b`) exhibits primary and secondary spermatocytes with severe nuclear anomalies, including karyolysis (green arrows), karyorrhexis combined with irregular nuclear envelopes (red circle), pyknosis (green circle), degenerated parietal cell (P), and lysis of the cytoplasm (asterisks). Fig. (c`) displays deteriorated secondary spermatocytes (SS). Fig. (d`) demonstrates a higher magnification of the inset in (c`), presenting the SS karyolysis (orange arrow), ruptured, and irregular nuclear envelope (Ne) (purple arrow).

To drill down the ultrastructure attributes of the testicular tissues obtained from the control and NiFe NPs-treated groups, TEM analysis was conducted. We thus inspected three different zones of development, involving growth, maturation, and differentiation. Examination of the growth zone of the control group revealed typical spermatogenic elemental structures. Spermatogonia were identified within the cysts with their characteristic large, rounded homochromatic nuclei and their noticeable nuclear envelopes surrounded by normal parietal cells as shown in Fig. 7a. In addition, Fig. 7b depicts the normal primary and secondary spermatocytes, differentiating by their large and small nuclei, respectively. Additionally, the nuclei of the spermatocytes were discerned in a round shape with homogenous chromatin and regular nuclear envelopes (Fig. 7c and d). On the contrary, the insects receiving NiFe NPs exhibited gradient and pronounced ultrastructural deformities as spermatogonia were noticed with manifest karyolysis and pyknotic nuclei, electron-dense vesicles, and vacuolations within the nucleoplasm as exhibited in Fig. 7a`. Moreover, primary, and secondary spermatocytes could be recognized with severe nuclear anomalies, including karyolysis, karyorrhexis, pyknosis, and irregular nuclear envelopes. Additionally, it could be detectable the lysis of the cytoplasm combined with disintegrated parietal cells along the developing cysts as portrayed in Fig. 7b`- d`.

**Fig. 8:**
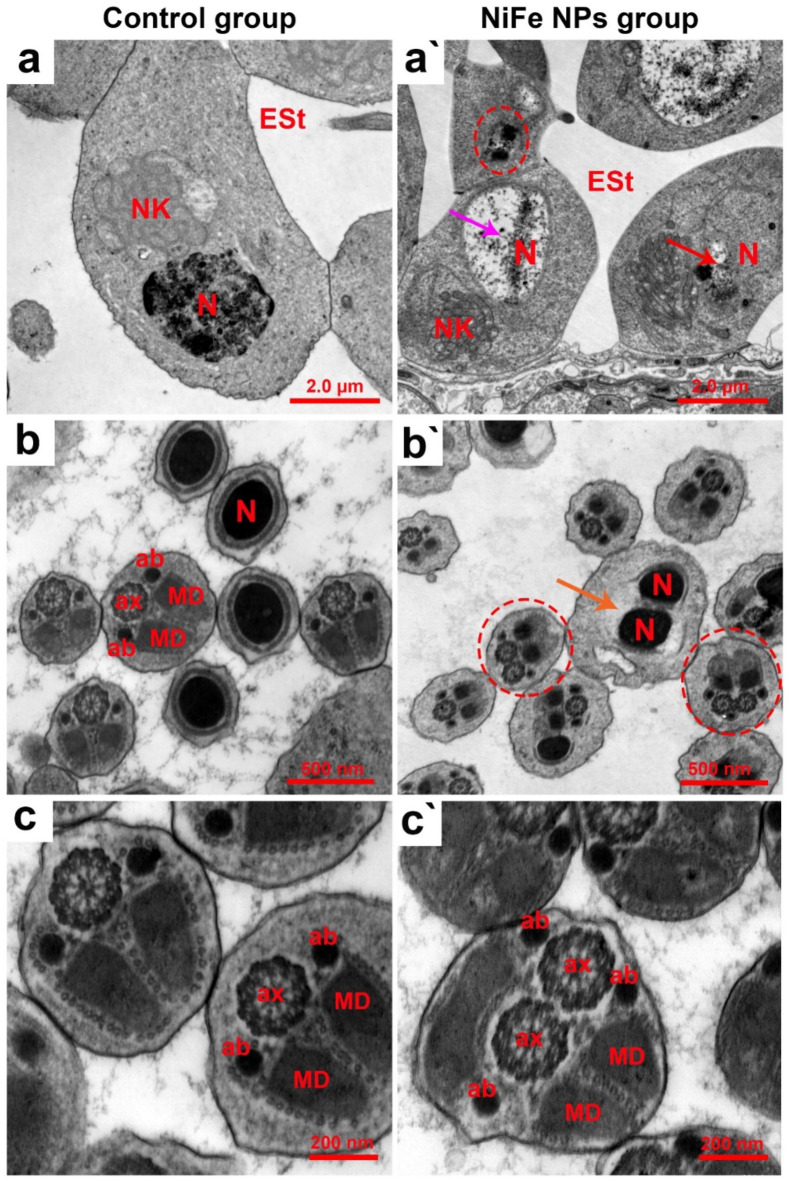
Ultrastructural analyses of the testicular maturation and differentiation zones of adult male *B. polychresta* dissected from control (a-c) and NiFe NPs-treated insects (a`-c`). Fig. (a) exhibits the typical structure of the early spermatid stage (Est), mitochondrial Nebenkern (NK), and nucleus (N). Fig. (b and c) reveal the distinctive spermatozoa heads and tails cross-sections, nucleus (N), and axoneme (ax), surrounded from each side by an accessory body (ab) and followed by a pair of mitochondrial derivatives (MD). Fig. (a`) exposes disintegrated mitochondrial Nebenkern (NK), degenerated nucleus (red arrow), pyknotic nucleus (encircled), and vacuolated nucleoplasm (purple arrow). Fig. (b`-c`) display the different spermatozoa anomalies, including cross-sections of double heads with paired nuclei (N) (orange arrow) or paired axonemes (ax) and three accessory bodies (ab) (encircled).

The control group’s maturation zone inspection revealed normal early spermatids, which are characterized by a prominent mitochondrial Nebenkern in front of a structurally preserved nucleus as shown in Fig. 8a. Additionally, the examination of the differentiation zone of the control beetles illustrated distinctive spermatozoa cross-sections of spherical, highly compacted nuclei and flagella consisting of a central axoneme surrounded from each side by an accessory body and followed by a pair of mitochondrial derivatives as illustrated in Fig. 8b and c. However, the inspection of the maturation zone of the testicular tissue harvested from the NiFe NPs-treated insects exhibited ultrastructural anomalies of early spermatids, including mitochondrial Nebenkern disintegration, degeneration, and apoptotic features of pyknosis, vacuolated nucleoplasm, and nuclear degeneration (Fig. 8a`). Besides, spermatozoa cross-sections from the group treated with NiFe NPs revealed various aberrations, including signs of either head or tail agglutinations. Cross-sections of double heads or paired axonemes with three accessory bodies within the same flagella cross-section were noticed as depicted in Fig. 8b`and c`.

## Discussion

The expansion of insecticidal applications has led to growing concern about insecticide resistance among agricultural pests. Therefore, the exploration of novel eco-friendly strategies, such as nanopesticides, is imperative to control various types of pests without hazardous effects on the environment^1,9^. However, the prolonged use, high mobility, and ecological toxicity of conventional nanopesticides have raised environmental concerns about their applications in agriculture. Therefore, green synthesized nanopesticides using biological sources provide sustainable solutions for agricultural productivity while safeguarding the environment^21^. This study was thus designed to fabricate new green synthesized NiFe NPs and investigate their potential insecticidal effects for the first time using the *B. polychresta* beetle as an insecticidal model. Toward this end, we assessed the influence of NiFe NPs as nanopesticides on testicular tissues of beetles through investigating pathophysiological, genotoxicity, and cellular toxicity, in addition to histopathological and ultrastructural alterations in testes.

In this study, we utilized lemon juice, which is a rich citrus source of ascorbic acid and citric acid for green synthesis fabrication of NiFe NPs. The availability and low cost of lemon make it an economical choice for fabricating NiFe nanoparticles on an industrial scale. Importantly, the lemon extract acts as a bio-reductant throughout the fabrication of NiFe NPs and other NPs like silver, copper ferrite, cobalt ferrite, etc^37,38^. Furthermore, this bio-assisted approach does not need harsh conditions, such as high pressure and temperature, and prevents using toxic and detrimental reagents^39,40^. Notably, green nanoparticles are required to remediate insecticide residuals in the environment to ensure sustainable agricultural applicability and outdo their harmful impacts on ecosystems, non-target species, and human health^21^.

Our findings demonstrated that the green synthesized NiFe NPs was successfully fabricated adopting the hydrothermal synthesis procedure. The XRD crystallography revealed the well-crystallinity of NiFe NPs, and the FTIR spectrum corroborated its structural composition. In addition, the morphological analysis of NiFe NPs exhibited their quasi-spheroidal morphology with a size ranging from 20.18 to 30.25 nm. The SEM and TEM images of NiFe NPs presented their aggregation state due to their ferromagnetic character with a magnetization value of 27.19 emu/g as demonstrated from the VSM hysteresis loop.

The application of biosynthesized NiFe NPs on adult *B. polychresta* at a single dose of 0.03 mg/g body showed the highest mortality in beetles, reporting 67% mortality after 48 h. By contrast, the mortality rates of beetles significantly dwindled with the increase of NiFe NPs doses. This could be related to the magnetic properties of NiFe NPs, which predominantly contribute to their accumulation within insect tissues^41^. Higher concentrations may increase the possibility of NiFe NPs agglomeration, resulting in their accumulation within a few tissues. However, circulating lower concentrations of NiFe NPs can contribute to a wide-ranging accumulation in multiple organs, affecting overall insect homeostasis and potentially resulting in death. Furthermore, it is preferable to use lower doses that do not kill an extremely large proportion of susceptible insects, which could benefit agriculture^42^. It has been reported that pesticide dosing and pesticide-induced stress can affect mutation rates, leading to major single-gene resistance or the accumulation of multiple mutations, which leads to multifactorial resistance. Crucially, insects may develop resistance to high pesticide doses due to increasing mutation rates. This is mainly due to the faster accumulation of nanoparticles, for instance, during pesticide administration^43^. Accordingly, we presume that beetles injected with high doses of NiFe NPs could adapt to nanoparticles. Interestingly, although the NiFe NPs were synthesized following a green synthesis approach implying their safety profile, such a low dose diminishes the chance of NiFe NPs toxicity to the environment, safeguarding environmental homeostasis.

Importantly, SEM-EDX analysis evinced the bioaccumulation of NiFe NPs in the testicular tissues of *B. polychresta* of treated beetles, revealing a significant accumulation of Ni and Fe compared to the control beetles. This implies the ability of NiFe NPs to traverse the blood-testis barrier of *B. polychresta* due to their size, provoking detrimental effects on the beetles with a remarkable lethal rate. Previous investigations demonstrated that a similar size range of nanoparticles could penetrate membrane barriers and accumulate in reproductive systems of insects, resulting in pernicious effects and high mortality rates^1,20,44,45^. Furthermore, our previous study demonstrated significant Ag accumulation in the testicular tissue of beetles exposed to Ag NPs^18^.

It is suggested that pathophysiological observations provide significant insights into determining the insecticidal intoxication of various insecticide agents, comprehending their mode of action, and interpreting the resistance development^17^. In this context, the response of cells to different insecticides is highly interacted through the activation of mechanisms to alleviate the physiological disturbances^46^. However, the failure of insects to regulate the stress triggered by insecticides implies the effectiveness of these agents. Thus, in order to evaluate the insecticidal property of NiFe NPs and decipher their mode of action against *B. polychresta*, we assessed the non-enzymatic and enzymatic oxidative stress markers in the testicular tissue homogenates of beetles, including MDA, GSH, SOD, CarE, HSP 70, and MT1 after exposure to a single dose of biosynthesized NiFe NPs. Oxidative stress incited by NiFe NPs led to critical deregulations in physiological properties of *B. polychresta*, which could be reported by augmentation of lipid peroxidation, inhibition of antioxidant enzymes, and DNA impairment, in addition to cellular necrosis and apoptosis.

It is broadly recognized that MDA is the by-product of lipid peroxidation that could strongly bind with DNA and proteins of insects, inducing DNA impairment and alterations in protein structures^18^. Lipid peroxidation also thwarts the activity of different biocatalysts and perturbs the function of transport proteins in the plasma membrane^47,48^. Our observation is in line with a previous report, which demonstrated that a noteworthy increase in MDA signifies the excessive production of ROS^49^. In a similar manner, a recent report demonstrated the generation of ROS following exposure to Fe NPs, leading to oxidative stress along with cellular impairments in insect pests^3^. It is worth mentioning that the amplification of MDA level implies NiFe NPs intoxication, explaining the elevated DNA damage observed in the comet assay and apoptotic cells. Correspondingly, a recent study showed a remarkable rise in MDA levels as a result of silkworm exposure to lead, engendering the apoptosis of cells in the intestinal epithelium^50^. On the other hand, significant reductions in the GSH and enzymatic activities of SOD and CarE as a result of beetles’ exposure to NiFe NPs point to disorder of the antioxidant defense system in beetles. To elaborate further, SOD plays a paramount role in abolishing oxidative stress inside cells^18^, and the lessening in SOD activity in this study reflects the failure of the testicular cells to detoxify superoxide O_2_^−^. In the same manner, GSH represents a key antioxidant through the scavenging of ROS^51^, in addition to its crucial role in hindering membrane lipid peroxidation^52^. Therefore, the reduction in GSH in NiFe NPs-treated beetles contributed to elevated lipid peroxidation by overproduced ROS. Moreover, the lower activity of CarE may refer to its malfunction in the detoxification process and the effectiveness of biosynthesized NiFe NPs in impeding the resistance of beetles. Similarly, previous investigations demonstrated that Ag NPs accumulation in cellular organelles could interfere with enzymatic activities, such as SOD and CarE, suggesting that silver ions induced critical alterations in detoxification enzymes^53^. Altogether, the antioxidant defense system of *B. polychresta* from NiFe NPs-treated beetles was severely disrupted due to its failure to control ROS overflow.

During the insect stress response, protein synthesis becomes energetically costly due to mitochondrial dysfunction, requiring excessive ATP^54^. Therefore, metal contamination and the high-stress response could adversely impact insect fitness^55^, including the upregulation of HSP70 and MT gene expression as reported in our current findings. Heat shock proteins (HSPs) are major responsive proteins for sustaining cell viability. Among HSPs, HSP70 is defined as the most vital molecular chaperone due to its indispensable role in cellular networking^46^. Under normal conditions, the expression of HSPs is crucial to maintaining cellular homeostasis^55^, explaining the appropriate level of HSP70 expressed in control beetles. Conversely, the upregulation of HSP 70 in testicular tissues obtained from beetles exposed to NiFe NPs suggests cells’ inability to maintain regular metabolic pathways, which agree with previous observations^46^. The sharp rise of HSP70 expression in NiFe NPs-treated beetles is likely related to a defensive strategy to reinstate cellular integrity. Additionally, a previous report exhibited that inhibition in SOD activity is correlated with elevation in HSP70 expression level in larvae of *Lymantria dispar* following their exposure to cadmium^55^. This could enhance their susceptibility to further stress, potentially reducing the overall number of fertilized eggs.

Beside the HSPs, metallothioneins (MTs) are crucial proteins implicated in metal detoxification and ROS homeostasis^56,57^. Their capacity to eliminate heavy metals and sequester untestable metal ions, as well as modulate essential metal concentrations, makes them vital components in preserving metal homeostasis and precluding metal-instigated toxicity^7,57^. Thus, the induction of MTs expression following metal exposure makes them a key biomarker for metal contamination and the triggered oxidative stress^58^. Due to the paramount role of MTs in insects, they typically express, but at an adequate level^54^, which justifies the low level of MT1 expression in the control insect in the current study. By contrast, the exposure to NiFe NPs engendered a remarkable growth in MT1 expression in the beetle’s testicular tissue. These observations are consistent with earlier findings, which showed increased MT levels in *Ostrinia furnacalis* subjected to Cd, Cu, and Zn^59^ and *Drosophila melanogaster* exposed to Pb^56^. Taken together, the increase in MT1 and HSP70 expression in testicular tissue exposed to biosynthesized NiFe NPs emphasized the deregulation in metabolic mechanisms in the testicular tissue of beetles.

Regarding the genotoxic and cytotoxic effects of biosynthesized NiFe NPs on beetles’ testicular cells, our results revealed substantial DNA impairment and augmentation in necrotic and apoptotic cells, associated with a reduction of viable cells in NiFe NPs-treated beetles. Previous observations support our findings, which evinced substantial DNA impairment and cellular toxicity correlated with dysregulation of the antioxidant defence system following intoxication with different metal NPs^20,44,60,62^. The interaction of biosynthesized NiFe NPs with beetles’ nucleic acid is likely related to their electrochemical manners toward nucleic acids, resulting in genotoxicity in testicular cells as previously elucidated^11^. It has been shown that the antioxidant impact of GSH can regulate cell death by mediating the main regulatory redox signaling pathway, indicating the pertinent link between GSH and cell death^51^. Furthermore, a previous study revealed oxidative stress induction through elevated ROS production and GSH depletion with apoptotic stimulation in human lung epithelial (A549) cells following treatment with NiFe NPs^63^. Consequently, GSH inhibition in our results may interpret its correlation with testicular cell death following exposure to NiFe NPs. Taking into consideration all these findings, we can unravel the underlying mechanisms of NiFe NPs as nanopesticides by unleashing surplus ROS, inciting oxidative stress. As a result, oxidative stress interfered with the antioxidant defense system, leading to DNA damage and cellular apoptosis. This hypothesis is corroborated by previous investigations, which demonstrated the toxicity mechanisms of different metallic nanoparticles^1,3,19^. Furthermore, this mode of action is similar to that exerted by Ag NPs, which induce the overproduction of oxidative stress biomarkers, including HSPs linked to protein synthesis, leading to toxicity and apoptotic cell death in insects^53^. Collectively, it could be inferred that NiFe NPs triggered oxidative injury in *B. polychresta* testicular tissue, resulting in male reproductive system dysfunction.

**Fig. 9:**
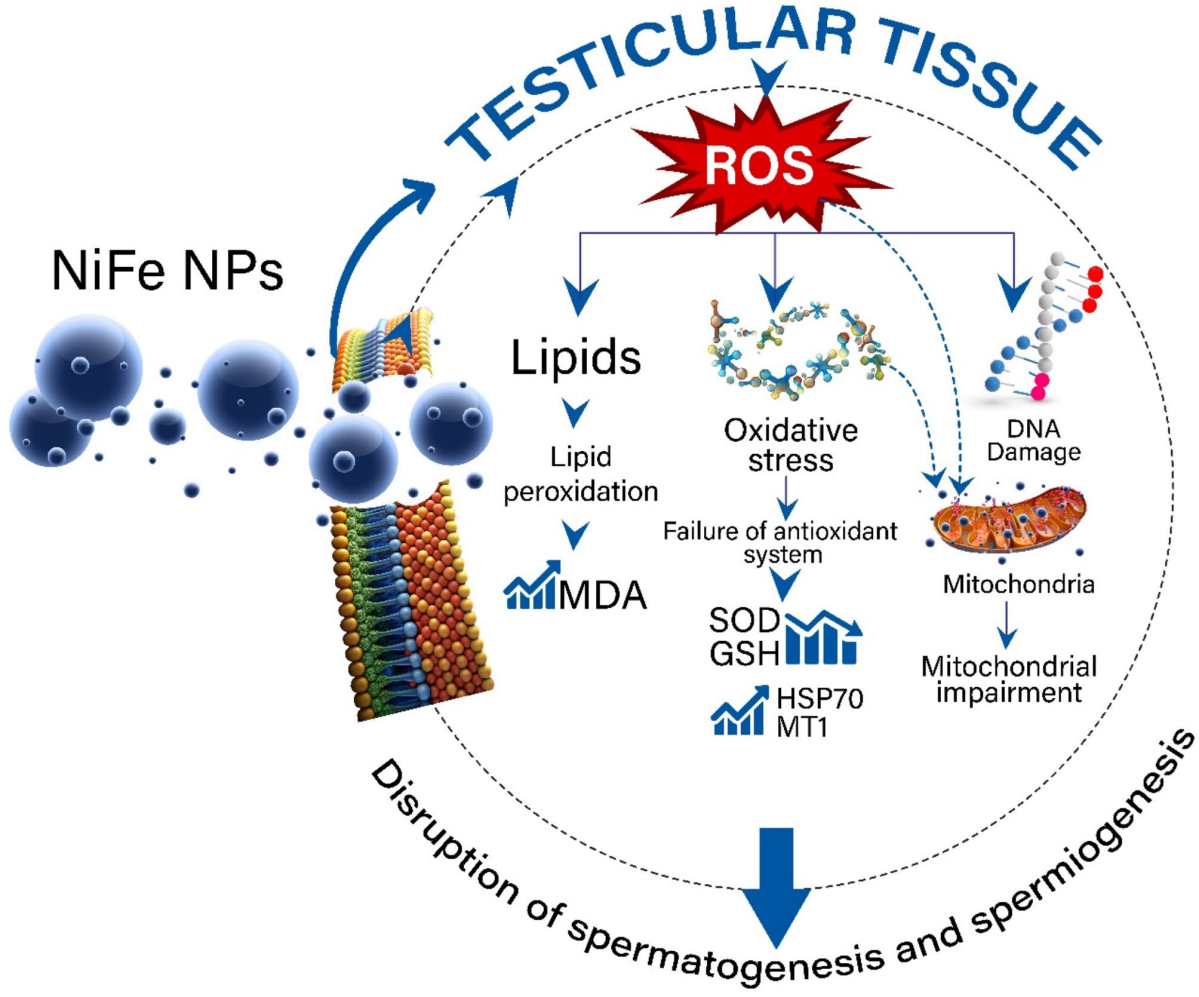
A schematic illustration reveals the insecticidal mechanism of the biosynthesized NiFe NPs within cells of testicular tissue of *B. polychresta*, provoking failure of spermatogenesis and spermiogenesis.

As anticipated, exposure of *B. polychresta* males to NiFe NPs resulted in noticeable aberrations in histological and ultrastructure analyses of testicular tissues. The most striking observations to emerge from histological examination are a reduction in cyst numbers, impaired follicular and cyst walls, deteriorated parietal cells, necrosis, vacuolations, and karyorrhexis. A comparable series of damages and necrosis in the insect’s spermatogenic elements was perceived due to the intoxication of metallic nanoparticles^18,20^. Based on TEM investigation, substantial effects of NiFe NPs were noticed on the ultrastructure of the spermatogenic elements across various development zones, such as spermatogonial karyolysis, pyknosis, electron-dense vesicles, and vacuolated nucleoplasm. Besides, corresponding anomalies were detected in spermatocytes. Additionally, early spermatids exhibited apoptotic features, including mitochondrial Nebenkern disintegration. Ultrastructural observations of the current study are in accordance with several previous studies that investigated the harmful impacts of heavy metal nanoparticles or chemical insecticides on insects’ testicular tissue^20,64,66^. Likewise, previous investigations evidenced ultrastructural malformations and membrane disruptions in coleopteran models after exposure to different insecticide toxicity^17^. In addition, our recent study reported that the bioaccumulation of Al_2_O_3_ NPs in the testicular tissue of migratory locusts, *Locusta migratoria*, led to manifested testicular abnormalities due to ROS overproduction^20^. It is worth mentioning that our biosynthesized NiFe Nps exhibit similar toxicity; however, due to their fabrication method, they are expected to be environmentally friendly. This argument is supported by recent findings of Tang et al.^67^, who reported the biocompatibility and low toxicity of NiFe NPs and their key role in enhancing maize *Zea mays* L. tolerance to abiotic stresses, increasing seed germination and seedling vigor. Overall, Fig. 9 depicts the proposed insecticide mechanism of NiFe NPs against *B. polychresta* in light of the findings presented in this study.

The compelling evidence provided in this study concerning the insecticidal effects of NiFe NPs encourages further studies to clarify the resistance mechanisms toward NiFe NPs by studying their long-term toxicity, susceptibility, potential environmental risks, and inter-generational transferability. In addition, the toxicity of NiFe nano-insecticides against other pests and their interaction with various biotic and abiotic stressors should be investigated using different formulations to reduce environmental risks. Accordingly, it is necessary to optimize their applicability to agricultural systems. Upcoming studies should explore the impact of NiFe NPs on other insects and load these nanoparticles onto various carriers for application to plants at a small scale in the lab before implementing them in the field. This could pave the way for sustainable and cost-effective insect pest management in agriculture.

## Conclusion

To sum up, this study demonstrated, for the first time, the insecticidal properties of the biosynthesized NiFe NPs against darkling beetles (*B. polychresta*) through accumulation in testicular tissues, disrupting the physiological and structural features of testes. The lowest and highest insecticidal dose of biosynthesized NiFe NPs was determined for sustainable and cost-effective management. Remarkably, the results revealed that NiFe NPs have the ability to pass through the *B. polychresta* blood-testis barrier, provoking detrimental effects on the beetles and even causing death. Exposure to NiFe NPs led to dysregulation of the antioxidant defense system associated with DNA damage and cellular apoptosis. Furthermore, these results are strikingly correlated with anomalies in testicular tissues in terms of ultrastructure features. These findings significantly contributed to deciphering the mode of action of NiFe NPs as nanopesticides, leading to dysfunctional male testicular tissues. Further investigations are required to examine the effect of NiFe NPs on other insect pests and evolve this approach for application in the field.

## Data Availability

The datasets used and/or analyzed during the current study are available from the corresponding author on reasonable request.
